# The Put-and-Fetch Ambiguity: How Magicians Exploit the Principle of Exclusive Allocation of Movements to Intentions

**DOI:** 10.1068/i0719sas

**Published:** 2015-04-01

**Authors:** Sander Van de Cruys, Johan Wagemans, Vebjørn Ekroll

**Affiliations:** Laboratory of Experimental Psychology, University of Leuven (KU Leuven), Leuven, Belgium; Laboratory of Experimental Psychology, University of Leuven (KU Leuven), Leuven, Belgium; Laboratory of Experimental Psychology, University of Leuven (KU Leuven), Leuven, Belgium

**Keywords:** border ownership, figure–ground, gestalt, magic, principle of exclusive allocation, ruse

## Abstract

In many magic tricks, magicians fool their audience by performing a mock action (a so-called “ruse”), which merely serves the purpose of providing a seemingly natural explanation for visible movements that are actually part of the secret move they want to hide from the audience. Here, we discuss a special magic ruse in which the action of secretly *putting* something somewhere is “explained away” by the mock action of *fetching* something from the same place, or *vice versa*. Interestingly, the psychological principles underlying the amazing potency and robustness of this technique seem to be very similar to the general perceptual principles underlying figure–ground perception and the assignment of border ownership. This analogy may be useful for exploring the possibility that this and similar magical effects involve immediate “unconscious inferences” about intentions more akin to perceptual processing than to explicit deliberations based on a reflective “theory” of mind.

Before reading on, you might want to view the excellent performance of Slydini's paperballs-to-hat routine available at https://www.youtube.com/watch?v=lvkRQgiwT0w (movie last accessed on 13 February 2015; description of the trick available in Milbourne, 2008, pp. 78-80). Here, the magician makes several paperballs disappear, and at the end of the performance they are mysteriously shown to be contained in a hat lying on the table, even though the hat was shown to be empty at the beginning of the performance. After viewing this performance, observers typically have no clue as to how the magician was able to get the balls into the hat.

Why do people usually have such a hard time figuring out the deceivingly simple secret behind this trick—which is that the magician *puts* the balls into the hat while pretending to *fetch* some invisible magic powder from it? Would it not be relatively easy to figure out that the magician can put something into the hat whenever he reaches into it?

We believe that an interesting analogy between this magical effect and the more well-known principles of figure–ground perception may point to an answer to this question. To appreciate this analogy, consider that when a magician moves his hand from A to B and back again (Figure 1a), this can, in principle be interpreted as three different intentional actions: He puts something from A to B (Figure 1b), he fetches something at location B and places it at A (Figure 1c), or he actually does both (Figure 1d). ^1^ Importantly, note the analogy to the perception of the central image contours in Rubin's (1915) famous face-vase demonstration (Figure 1c): In principle, they can be interpreted as reflecting three different real-world situations: A vase on a ground extending behind it (Figure 1f), two faces on a ground extending behind them (Figure 1g), or a mosaic of a vase-shaped object which happens to fit in snugly between two face-shaped objects (Figure 1h).

**Figure 1.**
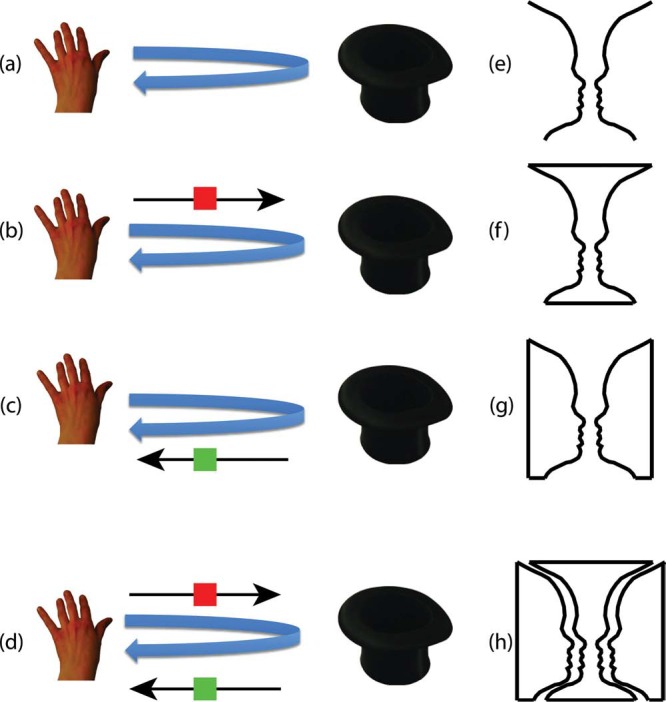
Illustration of the analogy between border ownership assignment in figure–ground perception and the assignment of “movement ownership” in the perception of actions. The contours in (a) are ambiguous and can be “owned” either by a vase (b) or two faces (c), or both at the same time (d). The visual system typically avoids the latter double assignment of border-ownership (“mosaic interpretation”) where the contours belong both to the vase and the faces (d). Analogously, the motion of the magician's hand into the hat and back is ambiguous in the sense that it may involve putting an object into the hat (f), fetching an object from the hat (g), or both at the same time (h). The latter double assignment of the “put” and “fetch” actions to the back-and-forth movement (h) seems to be avoided in much the same way as the assignment of double-border ownership in figure–ground perception (d). This could explain why we are so easily fooled by this kind of magical ruse.

In much the same way as the central borders can be “owned” either by the face or the vase, the back and forth movement of the magician's hand can be owned by a “putting action” or a “fetching action.” Importantly, research on figure–ground perception has shown that the visual system systematically avoids the double assignment of border ownership associated with the mosaic interpretation (Driver & Baylis, 1996). That is, it adheres to the general principle of exclusive allocation, according to which “a sensory element should not be used in more than one description at a time” (Bregman, 1994, p. 12). Thus, in the paperballs-to-hat routine, the sensory element (the actual back and forth movement), being allocated to the “mock” action of “fetching dust,” is not available for allocation to the action of putting things into the hat anymore.

In a more general sense, this kind of ambiguity, where a competitive interpretation is “eliminated” from awareness, characterizes the perception of bistable figures in general (Leopold & Logothetis, 1999; Tormey & Tormey, 1983). The surprising potency and robustness of the above magic trick can be elegantly explained by assuming that the visual system also adheres to this rule by avoiding the double assignment of “movement ownership” associated with the “put-and-fetch action” interpretation (Figure 1d): Our visual system simply won't produce more than one of the two potential action descriptions (“putting” or “fetching”) of the hand motion at the same time. As is well known, this general principle can be motivated by the fact that it tends to give right answers about how the visual input was actually generated (Albert & Hoffman, 1995; Bregman, 1994; Freeman, 1994) by avoiding interpretations involving unlikely coincidences: In the case of figure–ground perception, double-border ownership assignment would correspond to a highly unlikely jig-saw-puzzle-like alignment of the contours of two different surfaces, and in the case of action perception, the double assignment of movement ownership would correspond to the very unlikely situation where an object needs to be moved from A to B while another one also needs to be moved from B to A at the same time. The unlikeliness of this is precisely the reason why the magician has to come up with a mock action (or “ruse”), such as “fetching some magic dust from the hat,” or “getting the magic wand out of his pocket” (while actually ditching a “vanishing” coin, see https://www.youtube.com/watch?v=E6VWi7IaroA; movie last accessed on 13 February 2015).

At least since the seminal writings of Michotte (1950/1991) and Heider and Simmel (1944), scholars have noticed that high-level representations such as intentions attributed to motion can actually have strong perceptual qualities. Michotte (1950/1991) and Heider and Simmel (1944), demonstrated that people readily attribute animacy (intentions) and emotions to mechanical motion of simple geometric shapes. The automaticity and directness with which this happens surely suggest that the process underlying this is more akin to unconscious perceptual inference than to deliberate reasoning about likely intentions of the agent. In fact, *undoing* this requires deliberate thinking, as when trying to see only the proximal inputs of the apple without seeing the full apple (as in amodal volume completion, see Ekroll, Sayim, & Wagemans, 2013). Convergent evidence from phenomenology (Gallagher, 2008), neuroscience (Blakemore & Decety, 2001) and experimental psychology (Scholl & Gao, 2013) now supports the idea that any action is immediately perceived as having an intention or end goal, with a concomitant sense of visual presence.

One might ask why we inevitably perceive movements as intentions. The encoding of perceived movements in terms of end goals (intentions) attests to the predictive nature of perception (Van de Cruys & Wagemans, 2011). A perceived intention represents a movement more efficiently as part of a predictable chain of interdependent events, disregarding the fine details of the movements. Framing this within Bayesian or predictive models of perception shows that the exclusive allocation can also be considered a form of “explaining away” (Kersten, Mamassian, & Yuille, 2004). Specifically, if the same inputs (movement details) can be explained by two different hypotheses, heightening the probability of one explanation, e.g., by contextual cues, will automatically reduce the probability of the other hypothesis. To paraphrase the classic example for “explaining away”: the hypothesis that it just rained will be quasi-eliminated as an explanation for a wet lawn, once one notices a leaky garden hose lying around.

The present phenomenon can be described as one movement “overshadowing” the other in the spectator's mind: Either the forward “putting movement” overshadows the backward “fetching” movement, or *vice versa.* Many similar effects, where specific movements tend to overshadow others are well known to magicians (Kurtz, 1998). One general rule of thumb routinely used by magicians is that large movements tend to make smaller ones go unnoticed. In the paddle-move for instance, the large sweeping motion of a paddle (or any stick-like object) makes it very difficult to notice that it is also simultaneously rotated along its long axis (Hergovich, Gröbl, & Carbon, 2011). On a general level, all of these effects seem to work by making one of the movements more conspicuous than the other, whereby the latter can be manipulated relying on different factors. The present effect is probably no exception to this rule, but the cues that determine the relative dominance of the two possible interpretations are probably more subtle than the gross differences in motion size underlying the paddle move effect. Another important difference is that the paddle move involves two simultaneously performed movements, while the present effect involves a forward and a backward motion performed in succession. Furthermore, while it makes sense to speak of a fundamental ambiguity between the two (or strictly speaking, three) possible interpretations in the present case, the paddle move does not seem to involve any principled ambiguity, but rather seems to be driven by limits of the perceptual system (Hergovich et al., 2011).

In conclusion, we generalized the principle of exclusive allocation to action-intention perception and described how it is exploited in magic tricks. This case study underscores the usefulness of magic to uncover general principles in perception and cognition (Kuhn, Amlani, & Rensink, 2008).

## Footnotes

1 It may be of some interest to note that also a fourth interpretation is logically possible, namely that nothing is put or fetched at all, but just kept in the hand all the time. In terms of the analogy to border ownership, this may be said to correspond to a contour without surfaces on either side.
